# Post-Exertional Malaise May Be Related to Central Blood Pressure, Sympathetic Activity and Mental Fatigue in Chronic Fatigue Syndrome Patients

**DOI:** 10.3390/jcm10112327

**Published:** 2021-05-26

**Authors:** Sławomir Kujawski, Joanna Słomko, Lynette Hodges, Derek F. H. Pheby, Modra Murovska, Julia L. Newton, Paweł Zalewski

**Affiliations:** 1 Department of Hygiene, Epidemiology, Ergonomics and Postgraduate Education, Division of Ergonomics and Exercise Physiology, Collegium Medicum in Bydgoszcz, Nicolaus Copernicus University in Torun, 85-094 Bydgoszcz, Poland; jslomko@cm.umk.pl (J.S.); p.zalewski@cm.umk.pl (P.Z.); 2 School of Sport, Exercise and Nutrition, Massey University, Palmerston North 4442, New Zealand; l.d.hodges@massey.ac.nz; 3 Society and Health, Buckinghamshire New University (Retired), High Wycombe HP11 2JZ, UK; derekpheby@btinternet.com; 4 Institute of Microbiology and Virology, Riga Stradiņš University, LV-1067 Riga, Latvia; Modra.Murovska@rsu.lv; 5 Population Health Sciences Institute, The Medical School, Newcastle University, Newcastle-upon-Tyne NE2 4AX, UK; julia.newton@ncl.ac.uk

**Keywords:** PEM, myalgic encephalomyelitis, brain fog, vascular stiffness

## Abstract

Post-exertional malaise (PEM) is regarded as the hallmark symptom in chronic fatigue syndrome (CFS). The aim of the current study is to explore differences in CFS patients with and without PEM in indicators of aortic stiffness, autonomic nervous system function, and severity of fatigue. One-hundred and one patients met the Fukuda criteria. A Chronic Fatigue Questionnaire (CFQ) and Fatigue Impact Scale (FIS) were used to assess the level of mental and physical fatigue. Aortic systolic blood pressure (sBPaortic) and the autonomic nervous system were measured with the arteriograph and Task Force Monitor, respectively. Eighty-two patients suffered prolonged PEM according to the Fukuda criteria, while 19 did not. Patients with PEM had higher FIS scores (*p* = 0.02), lower central systolic blood pressure (*p* = 0.02) and higher mental fatigue (*p* = 0.03). For a one-point increase in the mental fatigue component of the CFQ scale, the risk of PEM increases by 34%. For an sBPaortic increase of 1 mmHg, the risk of PEM decreases by 5%. For a one unit increase in sympathovagal balance, the risk of PEM increases by 330%. Higher mental fatigue and sympathetic activity in rest are related to an increased risk of PEM, while higher central systolic blood pressure is related to a reduced risk of PEM. However, none of the between group differences were significant after FDR correction, and therefore conclusions should be treated with caution and replicated in further studies.

## 1. Introduction

Chronic Fatigue Syndrome (CFS) is characterized by a substantial deterioration of symptoms that could be provoked in response to physical exercise in patients with post-exertional malaise (PEM) [1]. The National Academy of Medicine reports the prevalence of PEM among CFS patients as being from 69 to 100% [2]. PEM is regarded as a hallmark symptom of CFS [3]. However, some criteria, such as the Fukuda case definition [4], do not require PEM to be present for a CFS diagnosis. The majority of patients, i.e., 73.4%, reported the duration of PEM as being equal to or longer than 24 h [5].

The exact mechanism that underlies PEM is as yet unknown. However, it has been reported that patients with CFS experience shortness of breath [6]. Disruption of the resting respiratory rate induced by PEM has been observed [7].

In addition, an attenuated adrenaline response to physical exercise has been observed in CFS, compared to healthy controls [8], along with more pronounced increases of nitric oxide metabolites after a physical exercise test [9]. The decreased response of adrenaline to physical exercise and a disturbance of nitric oxide metabolites are in line with the observations of Bond et al. [10], who recently proposed that a disturbance in the functioning of the vascular system was a key factor in PEM pathogenesis.

A decrease in the ability of large arteries to adapt readily to an increase in the amount of blood ejected during heart muscle contraction has been reported. Aortic pulse wave, or pulse wave propagation velocity (PWV), and an indirect parameter, the augmentation index (Aix), constitute a relatively simple, non-invasive, and reproducible method to determine arterial stiffness [11]. Arterial stiffness has been found to be associated with cardiovascular events in older cohorts, and, when measured, PWV is considered to be a significant risk factor for cognitive decline. Furthermore, a less elastic arterial system occurs together with impaired autoregulation of cerebral perfusion. In consequence, episodes of hypotension might lead to an increase in the risk of brain hypoperfusion. High arterial stiffness might occur in CFS patients [12] and could serve as a marker of increased cardiovascular risk in this population. Słomko et al. show that CFS patients with sympathetic autonomic dominance had the highest value of arterial stiffness, compared to patients with autonomic balance [13]. Hunter et al. suggest that a higher elasticity of large arteries was correlated with lower subjective fatigue in older women with CFS [14].

The Fukuda criteria can distinguish between CFS and chronic fatigue. However, a further subclassification of the former group into PEM positive and PEM negative subgroups has been suggested [15]. In contrast to the Fukuda criteria, the Canadian Consensus Criteria (CCC) require PEM for a diagnosis of CFS [16]. Therefore, it has been suggested that patients diagnosed on the basis of the Fukuda criteria are a heterogeneous group [17]. However, unlike generalized fatigue, PEM can be associated with extreme disruption of daily life functioning [18]. However, the Fukuda definition, which has been in use for twenty-seven years, has, until recently, been the most widely used case definition for ME/CFS, and is still very widely used in research and clinical practice. The CCC case definition identifies a more severely affected subgroup of those patients identified by the Fukuda definition. The main feature which serves to distinguish those Fukuda-positive patients who are also CCC-positive from those who are not is the presence of PEM. We decided to rely on the Fukuda criteria for CFS to examine the differences between the CFS subgroups of patients with and without PEM. The underlying mechanism of PEM is still not fully understood; therefore we conducted a cross-sectional study to explore the differences in selected physiological parameters and symptoms severity in CFS patients with PEM, compared with those without. The aim of the current study is to examine differences in CFS patients with and without PEM in indicators of aortic stiffness, autonomic nervous system function, and severity of physical and mental fatigue.

## 2. Materials and Methods

The current study took place from January 2013 to July 2018. The Ethics Committee, of the Ludwik Rydygier Collegium Medicum in Bydgoszcz, Nicolaus Copernicus University, Torun approved the study (KB 332/2013, date of approval: 25 June 2013). Written, informed consent was obtained from all the participants.

### 2.1. Enrolment

A group of 131 patients with CFS between 25 and 65 years of age were recruited via telephone, e-mail, and mass-media advertisements. The main enrolment criteria included: 

(1) Fukuda criteria (2) Fatigue Severity Scale score higher than 36 points and persistent fatigue for more than 6 months (3) had suffered from one or more of four additional symptoms: post-exertional malaise, impaired memory and/or concentration, unrefreshing sleep, headache, sore throat, tender lymph nodes (axillary or axillary), muscle or joint pain, (4) perceived fatigue could not be explained by an underlying condition. On inclusion, all CFS patients received a pre-test health state assessment: basic neurological, psychiatric, clinical examination, and had been referred by a general practitioner and by the neurology and psychiatry departments. The exclusion criteria included: (1) any indication of underlying illness, (2) medical condition explaining fatigue, (3) psychiatric disorders. Hospital Anxiety and Depression Scale (HADS) has been performed to assess anxiety (HADS_A) and depression (HADS_D) symptoms intensity [19]. Beck Depression Inventory (BDI-II) was used to examine depression symptoms intensity [20].

30 participants were excluded as they did not meet the Fukuda criteria (*n* = 10), had an underlying psychiatric illness (*n* = 13), or had another diagnosis, or fatigue was not the primary complain (*n* = 7). 

On the day of investigation all subjects were instructed to eat a light breakfast, and refrain from smoking, caffeine, alcohol consumption and vigorous physical activity. All tests were carried out in a chronobiology laboratory (soundproofed room without windows, temperature 22 °C, humidity 60%) and were performed at approximately the same time of day.

### 2.2. Measurement

#### Scales

The Chalder Fatigue Questionnaire (CFQ) [21], Fatigue Severity Score (FSS) [22] and the Fatigue Impact Scale (FIS) [23] were administered to provide a comprehensive assessment of fatigue severity. The CFQ assessed physical and psychological fatigue. It consists of 11 items that could be divided to mental (4 items) and physical fatigue (7 items) dimensions. Scoring was made in “Likert” style (in a range from 0 to 3) therefore the total score could be 0 at minimum and 33 at maximum and from 0 to 12 in mental and from 0 to 21 points in physical dimension. Moreover, binary scoring of the total score (0 for absence ad 1 for presence) was done. FSS assessed fatigue in the past week. It consists of nine items that are statements. Patients could choose an option from strongly disagreeing with a statement (1 point) to strongly agreeing with a particular statement (7 points). Total scores ranged from 9 to 63 points. FIS assessed cognitive, physical, and psychosocial fatigue. Higher scores indicate higher severity in all domains. Likert-like scoring with a range of 0–4 points per item was applied to 40 items in total. Therefore the total score could range from 0 to 160 points. In all fatigue questionnaires, the higher the results in points, the more severe fatigue.

The Hospital Anxiety and Depression Scale (HADS) was performed to assess anxiety (HADS_A) and depression (HADS_D) symptoms intensity [19]. The Beck Depression Inventory (BDI-II) was used to examine depression symptoms intensity [20]. Both scales were used only at the baseline to exclude patients with depression.

The Epworth sleepiness scale (ESS) [24] was used to assess patients’ general daytime sleepiness.

### 2.3. Autonomic Symptom Assessment

We used subjective and objective tools to measure the function of the autonomic nervous system. The Autonomic Symptom Profile served to measure presence, frequency, and dynamics of autonomic symptoms severity [25]. Scoring was done based on the Composite Autonomic Symptom Score 31 (COMPASS 31) [26]. Questionnaire contains 31 items assessing six dimensions of autonomic nervous system function, namely response to orthostatic stress, vasomotor and secretomotor reactions, function of, bladder and gastrointestinal tract, as well as pupillomotor reflex. Scores from individual domains were weighted. The total score is 100 points, the higher the score the higher the symptom load. In addition, Orthostatic Grading scale (OGS) was used to assess response to orthostatic stress [27].

Second, ANS functioning was automatically measured with a Task Force Monitor—TFM (CNS Systems, Gratz, Austria). Signals from a three-channel ECG were analyzed using the adaptive autoregressive model [28]. Low frequency (LFnu-RRI) (0.04–0.15 Hz) and high frequency (HFnu-RRI) (0.15–0.4 Hz) components of R-to-R intervals in normalized units, as well as its ratio (LF/HR-RRI), were recorded and analyzed in rest. Assessments were performed after a 5 min waiting period in a supine position, which allowed for signals to stabilize. Then, an assessment at rest was performed in supine position for a further 5 min.

### 2.4. Arterial Stiffness

Arterial stiffness was measured using an oscillometric non-invasive Arteriograph (TensioMed Kft, Budapest, Hungary, www.tensiomed.com, accessed on 18 March 2021). This is a device that uses a simple upper arm cuff as a sensor, with the cuff pressurized to at least 35 mmHg over the actual systolic pressure. The device determines the PWVaortic and augmentation index according to the manufacturer’s instructions. Arteriograph measurement has been described extensively in previous papers [29,30,31]. The Arteriograph, simultaneously with the arterial stiffness parameters, also records the actual systolic and diastolic blood pressure (BPs) and heart rate.

The difference between the central and peripheral systolic blood pressure was calculated based on measurement from arteriography and TFM, respectively.

### 2.5. Statistical Analysis

Histogram visual inspection and the Shapiro–Wilk test were applied to test the normality assumption. To examine between-group differences (patients with PEM vs. those without) Mann–Whitney U or independent *T*-tests were used, depending on assumptions met. To predict presence of PEM in examined patients, a logistic regression model using the GLM function was applied in R. In addition, 95% confidence intervals for log-likelihoods and odds ratios were calculated (confit function using bootstrap). The DescTools package was used to calculate pseudo R2 for the model [32]. Dotwhisker plots were used to visualize odds ratios and confidence intervals [33]. Violin graphs were created using R [34] with a ggstatsplot library [35]. Effect sizes from the ggstatsplot library are reported for between group comparisons. The false discovery rate (FDR) was controlled using a Benjamini–Hochberg adjusted *p*-value, applying an online calculator available at (https://tools.carbocation.com/FDR, accessed on 18 March 2021). The results contain *p*-values before as well as after correction.

## 3. Results

One hundred and one patients met the Fukuda criteria for CFS and were included in the analysis (Table 1). The patients were divided into groups with PEM or without PEM. Eighty-two patients reported prolonged PEM, whilst nineteen patients were free of prolonged PEM. Both groups consisted predominantly of women, comprising fifty-one patients from PEM group (62.2%), and fourteen (73.7%) in the group without PEM. Table 1 describes detailed characteristics about the total group and group differences between the patients with PEM and those without. 

Table 2 presents differences in arteriography results between patients with PEM and those without PEM. Table 3 indicates differences between patients with PEM and those without PEM, in respect of autonomic nervous system function indicators.

Patients with PEM had higher overall fatigue, as measured by FIS (81.61 ± 30.3 vs. 63.05 ± 33.9, *t* = −2.19, *p* = 0.02, Hedges’ g = −0.57 (−1.07, −0.03), and higher mental fatigue (9.13 ± 1.8 vs. 7.95 ± 2.2, Z = 2.18, *p* = 0.03, r = −0.22 (−0.39, −0.03) (Table 1). Mean fatigue scores in the total sample were 23.68 points (range 0–33 points) in the CFQ scale, 47.03 points (range 9–63 points) in FSS, and 78.12 points (range 0–160 points) in FIS.

Patients with PEM had lower central systolic blood pressure than those without PEM (127.49 ± 15 vs. 141.05 ± 21.1, Z = −2.37, *p* = 0.02, r = 0.24 (0.06, 0.44) (Table 2). No significant differences in blood pressure measured peripherally were observed (Table 2).

Patients with PEM had higher LFnu-RRI (56.61 ± 16.7 vs. 46.64 ± 13.9, Z = 2.58, *p* = 0.01, r = −0.26 (−0.45, −0.06), lower HFnu-RRI (43.39 ± 16.7 vs. 53.36 ± 13.9, Z = -2.58, *p* = 0.01, r = 0.26 (0.07, 0.44) and higher LF/HF-RRI (1.96 ± 2.2vs. 1.13 ± 0.9, Z = 2.37, *p* = 0.02, r = −0.24 (−0.42, −0.06). Also, LF/HF was higher in patients with PEM (1.66 ± 1.5 vs. 0.94 ± 0.5, Z = 2.96, *p* = 0.003, r = −0.29, (−0.45, −0.14). In addition, patients with PEM had higher LFnu-dBP (53.88 ± 14.4 vs. 43.45 ± 13.8, Z = 2.69, *p* = 0.01, r = −0.27 (−0.47, −0.11) and higher LF/HF-dBP (7.27 ± 6.0 vs. 4.32 ± 3.6, Z = 2.36, *p* = 0.02, r = −0.23, (−0.45, −0.04) (Table 3).

**Figure 1:**
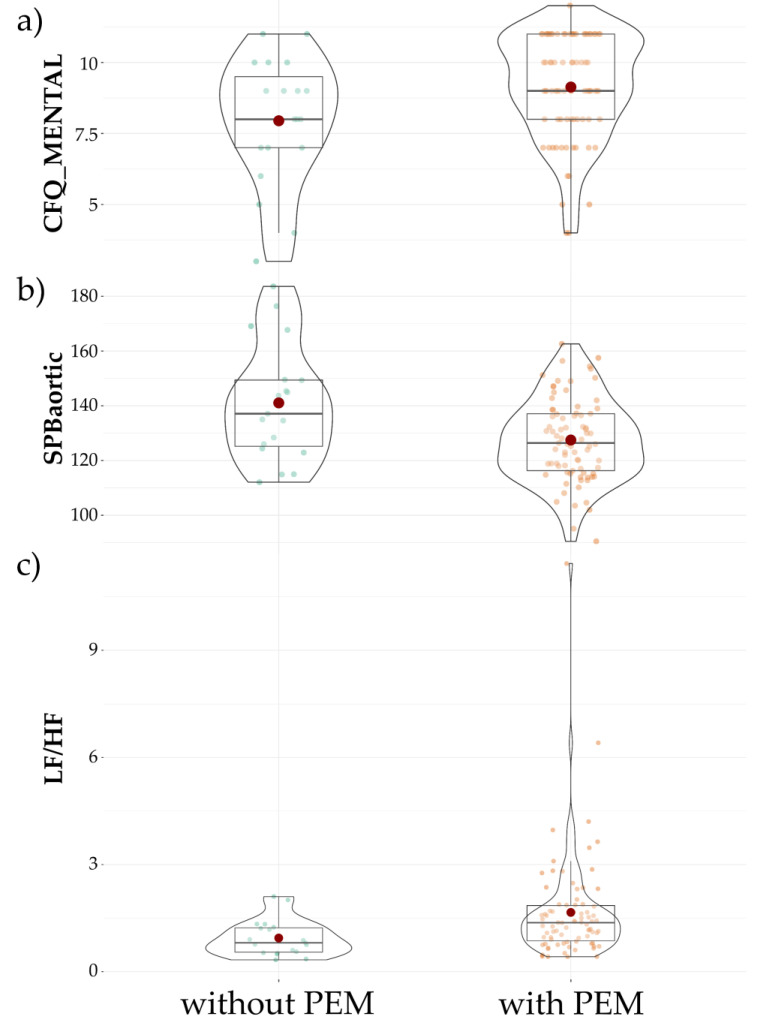
Comparison of patients with PEM vs. without PEM in the CFQ mental fatigue sub-score (**a**), aortic sBP (**b**) and sympathovagal balance (**c**). Red dots indicate mean value, horizontal black line inside the box denotes median value. Green dots before and orange dots after denote results of individual patients. Shape of violin graph indicates distribution of results.

Figure 1 presents differences between patients without PEM and patients with PEM in the level of the CFQ mental fatigue sub-score (Figure 1a), aortic sBP (Figure 1b) and sympathovagal balance (Figure 1c). This set of variables was then included in logistic regression models as PEM presence predictors.

**Figure 2:**
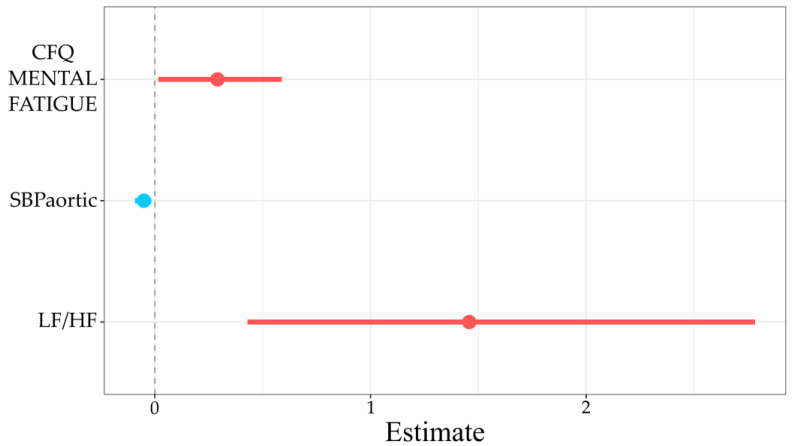
Logistic regression estimates predicting PEM presence. The horizontal axis refers to the odds ratio. Dots denote parameter estimates, while the horizontal lines through the dots denote 95% confidence intervals of the estimates.

Figure 2 shows the estimate of parameters and their confidence interval (−95%, 95%) in logistic regression analysis. In terms of odds ratios, the results were as follows: for a one point more increase in the mental fatigue component of the CFQ scale, the risk of PEM increases by 34% (CI = 2%, 80%). For an aortic systolic blood pressure increase of 1 mmHg, the risk of PEM decreases by 5% (CI = 9%, 2%). For a one unit more increase in sympathovagal balance, the risk of PEM increases by 330% (CI = 50%, 1516%). (AIC = 81.18, BIC = 91.6, Tjur’s *R*^2^ = 0.26) (Table A1 in Appendix A).

## 4. Discussion

The results of the present study have shown that CFS patients with PEM had significantly higher mental fatigue, overall fatigue being measured by FIS, which was one of the three fatigue scales used in the above research. Moreover, patients with PEM had lower central systolic blood pressure. However, no difference in levels of peripheral blood pressure was observed. Higher mental fatigue is related to a higher risk of PEM, while higher central systolic blood pressure is related to lower risk of PEM. However, none of the between group differences were significant after FDR correction, and therefore conclusions should be treated with caution and replicated in further studies.

As has been noted in the introduction, 73.4% assessed PEM duration as equal to or longer than 24 h [5]. As a result of the dramatic decline in patients’ physical and/or cognitive functioning, some patients might have to adjust their lifestyle and activity levels to avoid inducing PEM [2]. Some 25–29% of patients are reported to be bedbound or housebound [2]. The annual cost of CFS in the USA is estimated to be around $18 to $24 billion [2]. PEM was proposed as a prognostic indicator of CFS course [2], so studies of PEM have high clinical significance. Exploration of the PEM mechanism could lead to more effective therapeutic approaches, and thus to improvement of overall patient function.

The above findings are in line with previous research, which suggests that chronic vascular damage might cause a lack of exercise-induced vasodilation and be a potential cause of PEM [10]. In CFS, reduced blood pressure is frequently reported [36,37]. Additionally, an inverse relationship between increasing fatigue and diurnal blood pressure variation has been observed [38]. In our sample, despite a lack of difference in peripheral systolic blood pressure (with a mean lower than 120 mmHg in both groups), increased central systolic blood pressure was observed in patients with PEM. In certain conditions, such as during physical activity, the correlation between peripheral and central systolic blood pressure may decrease [38]. It is tempting to speculate that an increase in central systolic blood pressure in some CFS patients might occur before other pathological changes. Conversely, increased arterial stiffness was observed in healthy young adults in response to acute sleep deprivation. Arterial stiffness might therefore be secondary to comorbidities, such as sleep disturbance [39]. Further studies should examine the exact mechanism underlying the relationship between central systolic blood pressure, PEM fatigue, and factors responsible for blood pressure regulation in CFS. As a result of the rich symptomatology, an attempt should be made to classify different subgroups of CFS patients according to comorbidities. Further studies could possibly focus on the vascular system in all CFS patients, or perhaps just in a specific sub-group. Further exploration of this field would be helpful to establish possible personalized therapeutic approaches in CFS. In the present study, PEM presence was related to a higher mental fatigue component. Moreover, our previous study has shown that arterial stiffness parameters are related to fatigue severity; higher FIS and lower FSS scores were related to lower aortic stiffness [13]. Presumably, some of the mechanisms underlying this relationship could similarly link central blood pressure to PEM. Thus, mental fatigue might be related to dysfunction in ß2 adrenergic receptor and vascular or endothelial function, which in turn could lead to reduced cerebral blood flow (CBF) [40]. A decrease in CBF has been observed in CFS patients [41] and CBF has been shown to be correlated with fatigue severity [42]. Significantly, a negative correlation was seen between CBF and skeletal muscle pH at rest, which might explain why mental fatigue could be related to muscular fatigue [43]. However, the mechanisms described above are purely speculative and need to be examined in further studies.

In the current study, sympathetic nervous system function was more active during rest in the group with PEM, compared with the group without PEM. Moreover, indicators of parasympathetic nervous system activity were lower during rest. Findings in the PEM group are in line with the previous study by Frith et al. [36], where LF-dBP at rest was higher in a CFS group compared to controls. Parasympathetic activity was reduced in the CFS patients after inducing PEM [44]. During physical exercise, cardiac output distribution is governed by the sympathetic nervous system [45]. Surprisingly, in the PEM group, increased sympathetic drive coexisted with lower central systolic blood pressure. It is noteworthy that numerous factors can have an impact on the control of the ANS in the constriction of vascular beds, individual vessels, and even different parts of the same vessel [45]. Those factors include density and subtypes of adrenergic receptors and action of contransmitters, norepinephrine kinetics, density of sympathetic innervations, and degree of basal tone [45]. Additionally, local factors, such as the concentrations of vasoactive tissue metabolites, vessel size, and structure may also play a part in changes in vessel diameter and therefore in cardiac output distribution [45]. The disturbance in some of those factors could play an important role in PEM pathogenesis. Similar mechanisms for PEM have been proposed [40]. However, based on our findings, it is not possible to delineate the exact mechanism underlying the observed disturbances in ANS and whether it is related to PEM. Further studies should examine vascular function in response to stressors and its role in PEM. Understanding PEM mechanisms could contribute to the development of specific PEM therapy.

What is surprising is that the current results, corrected for multiple comparisons, showed no significant differences between the subgroup with PEM and that without PEM, in terms of fatigue as measured by scales. This is in contrast to a previous study, where higher PEM intensity was related to higher frequency and intensity of symptoms, and greater problems with emotional regulation [17]. However, discrepancies in the results observed might be related to a difference in methodology. In the current study, the division of CFS patients into PEM present or PEM absent subgroups was based on a binary variables (presence of prolonged post-exertional malaise), while May et al. divided patients into a group with no to moderate intensity of PEM (loPEM), and a group with severe to very severe PEM (hiPEM) [17]. Future studies should further examine if PEM is a distinct characteristic feature, on which basis a subgroup of CFS patients might be distinguished, or whether PEM is related directionally or non-directionally to other symptoms from which CFS patients suffer. In addition, it was suggested that further multi-center studies on CFS should apply the same protocols [46]. Therefore, it is necessary to make a decision as to whether PEM should be a symptom required for CFS diagnosis, which would have an impact on the types of criteria of CFS used in research. 

### Study Limitations

In the above study, PEM was measured subjectively. Further studies should incorporate a protocol based on repeated Cardiopulmonary Exercise Tests, to confirm objectively the presence of PEM in CFS patients. A difference in sample size between the subgroup with PEM and that without PEM was observed, which seems to reflect PEM prevalence in the general population. By increasing the sample size, a higher number of patients without PEM could be included as a control group. Sample size should therefore be increased in further studies on PEM in CFS. Meta-analysis showed that PEM occurs 10.4 times more frequently in CFS patients in comparison with other groups [47]. Comparison between patients with CFS might potentially reduce potentially confounding factors. The present study design is cross sectional, so no conclusions about causality can be drawn based on the current results. Further studies should explore the PEM mechanism applying intervention-based protocols. In addition, to reveal potential biological mechanisms, a deep phenotyping approach could be chosen, using a repeated CPET protocol to elicit PEM and to examine changes in biomarkers related to changes in PEM.

## 5. Conclusions

Patients with PEM had significantly higher mental fatigue and overall fatigue than those without PEM, as measured by FIS, one of the three fatigue scales used in the above research. They also had higher mental fatigue and sympathetic activity compared with parasympathetic activity during rest and lower central systolic blood pressure. However, none of these differences remained statistically significant after correction for multiple comparisons. Further studies should be conducted to confirm if higher mental fatigue and higher sympathetic activity, compared with parasympathetic activity at rest and lower central systolic blood pressure, are related to a higher risk of PEM.

## Figures and Tables

**Table 1:** Demographic data of total examined sample (*n* = 101) and comparison of demographic data and questionnaires results of patients with PEM and without PEM, respectively.

Variable	Total Sample	PEM Mean ± SD	Without PEM Mean ± SD	*p*-Value	FDR *p*-Value
Age [years]	38.15 ± 8.0	38.23 ± 8.1	37.79 ± 8.0	0.83	0.88
Height [cm]	171.55 ± 8.4	172.07 ± 8.7	169.32 ± 6.6	0.20	0.15
Weight [kg]	72.22 ± 12.6	72.77 ± 12.6	69.84 ± 12.5	0.36	0.61
BMI	24.47 ± 3.6	24.51 ± 3.5	24.31 ± 3.8	0.83	0.85
Symptoms duration [years]	4.54 ± 4.1	4.75 ± 4.2	3.64 ± 3.7	0.20	0.44
CFQ [points]	23.68 ± 4.6	24.07 ± 4.6	22.00 ± 4.4	0.08	0.21
CFQ_BINARY [points]	14.06 ± 4.8	14.49 ± 4.6	12.21 ± 5.1	0.07	0.22
CFQ_PHYSICAL [points]	10.47 ± 3.9	10.39 ± 4.0	10.79 ± 3.7	0.50	0.69
CFQ_MENTAL [points]	8.91 ± 2.0	9.13 ± 1.8	7.95 ± 2.2	0.03	0.11
FSS [points]	47.03 ± 10.1	47.57 ± 9.4	44.68 ± 12.8	0.46	0.68
FIS [points]	78.12 ± 31.6	81.61 ± 30.3	63.05 ± 33.9	0.02	0.09
HADS_A [points]	9.27 ± 3.5	9.22 ± 3.4	9.47 ± 4.2	0.78	0.87
HADS_D [points]	7.85 ± 3.4	8.00 ± 3.5	7.21 ± 2.9	0.36	0.63
BDI [points]	15.83 ± 7.9	16.15 ± 8.1	14.39 ± 6.9	0.57	0.75
ESS [points]	10.86 ± 5.6	11.01 ± 5.5	10.21 ± 6.3	0.65	0.78
OGS [points]	3.64 ± 3.2	3.71 ± 3.4	3.37 ± 2.5	0.98	0.98
Orthostatic intolerance [points]	11.96 ± 11.0	12.24 ± 11.3	10.74 ± 10.0	0.67	0.77
Vasomotor [points]	0.83 ± 1.4	0.86 ± 1.4	0.70 ± 1.4	0.57	0.73
Secretomotor [points]	5.52 ± 3.9	5.64 ± 3.9	4.96 ± 3.9	0.49	0.70
Gastrointestinal [points]	5.09 ± 4.2	4.89 ± 4.1	5.97 ± 4.7	0.37	0.60
Bladder [points]	0.54 ± 0.9	0.57 ± 1.0	0.41 ± 0.8	0.60	0.74
Pupillomotor [points]	1.15 ± 1.2	1.22 ± 1.2	0.86 ± 0.9	0.38	0.59
Compass-31 Total [points]	25.09 ± 14.7	25.43 ± 15.0	23.64 ± 13.4	0.81	0.88

BMI—body mass index, CFQ—Chalder Fatigue Questionnaire, FSS—Fatigue Severity Scale, FIS—Fatigue Impact Scale, HADS_A—Hospital Anxiety and Depression Scale anxiety, HADS_D—Hospital Anxiety and Depression Scale depression; BDI—Beck Depression Inventory, ESS—*Epworth Sleepiness Scale*, OGS—Orthostatic Grading Scale, Compass-31—Composite Autonomic Symptom Score 31.

**Table 2:** Comparison of arteriography results of patients with PEM and without PEM.

Variable	PEM Mean ± SD	Without PEM Mean ± SD	*p*-Value	FDR *p*-Value
PWVaortic [m/s]	8.33 ± 1.7	8.65 ± 1.8	0.21	0.41
Aixaortic [%]	28.11 ± 14.4	32.95 ± 15.5	0.20	0.41
sBPaortic [mmHg]	127.49 ± 15	141.05 ± 21.1	0.02	0.12
central-peripheral sBP [mmHg]	9.56 ± 21.7	23.41 ± 25.7	0.06	0.20

PWVaortic—Pulse Wave Velocity aortic, sBPaortic—aortic systolic blood pressure, central-peripheral sBP—difference between central and peripheral systolic blood pressure levels.

**Table 3:** Comparison of autonomic parameters in patients with PEM and without PEM.

Variable	PEM Mean ± SD	Without PEM Mean ± SD	*p*-Value	FDR *p*-Value
Spectral analysis of HR variability				
LFnu-RRI	56.61 ± 16.7	46.64 ± 13.9	0.01	0.12
HFnu-RRI	43.39 ± 16.7	53.36 ± 13.9	0.01	0.19
LF/HF-RRI	1.96 ± 2.2	1.13 ± 0.9	0.02	0.08
LF/HF	1.66 ± 1.5	0.94 ± 0.5	0.003	0.11
Spectral analysis of BP variability				
LFnu-dBP	53.88 ± 14.4	43.45 ± 13.8	0.01	0.09
HFnu-dBP	11.67 ± 7.3	18.20 ± 16.0	0.07	0.20
LF/HF-dBP	7.27 ± 6.0	4.32 ± 3.6	0.02	0.11
LFnu-sBP	42.86 ± 14.0	38.14 ± 13.7	0.19	0.44
HFnu-sBP	14.86 ± 9.3	18.74 ± 14.0	0.31	0.57
LF/HF-sBP	4.10 ± 2.8	3.02 ± 1.8	0.17	0.42

LFnu—low frequency normalized units, HFnu—high frequency normalized units, LF/HF—ratio of low frequency to high frequency, RRR—R to R interval, sBP—systolic blood pressure, dBP—diastolic blood pressure.

## Data Availability

Individual data is available from the corresponding author S.K. on request.

## Appendix A

**Table A1:** Logistic regression predicting PEM presence.

Term	Estimate	−95% CI	95% CI	*z* Value	*p*-Value
CFQ_MENTAL_FATIGUE	0.29	0.02	0.59	2.02	0.04
SBPaortic	−0.05	−0.09	−0.02	−2.66	0.01
LF/HF	1.46	0.43	2.78	2.45	0.01
